# Ligand-specific sequential regulation of transcription factors for differentiation of MCF-7 cells

**DOI:** 10.1186/1471-2164-10-545

**Published:** 2009-11-20

**Authors:** Yuko Saeki, Takaho Endo, Kaori Ide, Takeshi Nagashima, Noriko Yumoto, Tetsuro Toyoda, Harukazu Suzuki, Yoshihide Hayashizaki, Yoshiyuki Sakaki, Mariko Okada-Hatakeyama

**Affiliations:** 1 Cellular Systems Modeling Team, Computational Systems Biology Research Group, Advanced Computational Sciences Department, RIKEN Advanced Science Institute, 1-7-22 Suehiro-cho Tsurumi-ku Yokohama, Kanagawa, 230-0045 Japan; 2 Bioinformatics and Systems Engineering Division, RIKEN Yokohama Institute, 1-7-22 Suehiro-cho Tsurumi-ku Yokohama, Kanagawa, 230-0045 Japan; 3 RIKEN Omics Science Center, RIKEN Yokohama Institute, 1-7-22 Suehiro-cho Tsurumi-ku Yokohama, Kanagawa, 230-0045 Japan; 4 Toyohashi University of Technology, 1-1 Hibarigaoka, Tempaku-cho, Toyohashi, Aichi, 441-8580, Japan; 5 Cellular Systems Modeling Team, Research Center for Allergy and Immunology, RIKEN Yokohama Institute, 1-7-22 Suehiro-cho Tsurumi-ku Yokohama, Kanagawa, 230-0045 Japan

## Abstract

**Background:**

Sharing a common ErbB/HER receptor signaling pathway, heregulin (HRG) induces differentiation of MCF-7 human breast cancer cells while epidermal growth factor (EGF) elicits proliferation. Although cell fates resulting from action of the aforementioned ligands completely different, the respective gene expression profiles in early transcription are qualitatively similar, suggesting that gene expression during late transcription, but not early transcription, may reflect ligand specificity. In this study, based on both the data from time-course quantitative real-time PCR on over 2,000 human transcription factors and microarray of all human genes, we identified a series of transcription factors which may control HRG-specific late transcription in MCF-7 cells.

**Results:**

We predicted that four transcription factors including EGR4, FRA-1, FHL2, and DIPA should have responsibility of regulation in MCF-7 cell differentiation. Validation analysis suggested that one member of the activator protein 1 (AP-1) family, *FOSL-1 *(FRA-1 gene), appeared immediately following *c-FOS *expression, might be responsible for expression of transcription factor *FHL2 *through activation of the AP-1 complex. Furthermore, RNAi gene silencing of *FOSL-1 *and *FHL2 *resulted in increase of extracellular signal-regulated kinase (ERK) phosphorylation of which duration was sustained by HRG stimulation.

**Conclusion:**

Our analysis indicated that a time-dependent transcriptional regulatory network including c-FOS, FRA-1, and FHL2 is vital in controlling the ERK signaling pathway through a negative feedback loop for MCF-7 cell differentiation.

## Background

The activation, duration and subsequent inactivation of the extracellular signal-regulated kinase (ERK) signaling pathway triggers the induction of appropriate changes required for the determination of cell fate. Although many growth factors show remarkably similar profiles in terms of immediate early gene (IEG) mRNA expression in various cell lines [1-8], the biological outcome in response to these factors can vary. In fibroblasts, sustained ERK activation induced by platelet derived growth factor (PDGF) results in S-phase entry, unlike the case following transient activation by epidermal growth factor (EGF) [9-12]. *c-FOS *is a representative IEG which is expressed within minutes following stimulation with growth factor independent from duration of the upstream signals. However, the protein expression level of c-FOS is post-transcriptionally regulated by ERK activation kinetics, where transient ERK signals induced by EGF resulted in little induction of c-FOS, whereas sustained ERK activation by PDGF induced markedly higher amounts of the same protein [13,14]. Thus, the IEG product c-FOS can act as a molecular sensor for upstream ERK signals that leads cells towards particular paths such as proliferation, transformation or differentiation [13-15].

c-FOS is a member of the activator protein 1 (AP-1) transcription factor group that consist of FOS family proteins (c-FOS, FOSB, FRA-1/FOSL-1 and FRA-2/FOSL-2) [16-20] and JUN family proteins (c-JUN, JUNB and JUND) [21-23]. The AP-1 complex is formed by homo- and heterodimerization of JUN, FOS and several CREB/ATF family transcription factors, and mediates a wide range of biological effects related to cell growth, apoptosis and differentiation. c-FOS possesses the FXFP consensus motif, an ERK binding site, referred to as a DEF domain, which plays an important role as a sensor for ERK activity in cell fate decision [13,15,24]. In NIH 3T3 cells, mutation of the c-FOS DEF domain significantly reduced AP-1 activity and inhibited the transforming activity associated with wild-type c-FOS [13], suggesting that AP-1 transcription factor contains a DEF domain like c-FOS and that sustained ERK signals largely contribute to the regulation of cellular phenotypes.

We previously described that heregulin (HRG) induced sustained signal activity in MCF-7 breast cancer cells which triggered an irreversible cell phenotype change to differentiation (accumulation of lipid droplets within the cells), while EGF could only elicit transient signal activity and cell proliferation [25]. Notwithstanding differences in phenotype induction, EGF and HRG induced qualitatively similar early transcription profiles independent of the presence or absence of prolonged ERK signal activity, resulted in all-or-none induction of c-FOS protein [25]. Therefore, we assumed that the induction of different cell fates would eventually be accompanied by differences in the later transcribed genes after appearance of c-FOS. Ultimately, early quantitative differences in gene expression might be followed by qualitative differences during a series of transcriptional events. Therefore, in the current study we set out to identify phenotype-specific genes following long-term exposure to growth factors using time-course large-scale gene expression and to validate the function of the targeted genes. Expression analysis was first analyzed by quantitative real-time PCR (qRT-PCR) aimed at identifying core regulatory transcription factors. The results showed that ligand-specific transcription factors showing distinct expression are all induced by HRG but not by EGF.

Experimental validation was performed in an effort to confirm the late-transcriptional regulation of those transcription factors, c-FOS, FRA-1 and FHL2. Our results suggested that a time-dependent transcriptional regulatory network and associated control of the upstream signal activity by transcriptional feedback facilitate the entry of cells into an irreversible state.

## Methods

### Cell culture

The MCF-7 cell line was maintained in DMEM medium (Gibco BRL, Githersburg, MD) supplemented with 10% fetal bovine serum. Prior to growth factor treatment, cells were serum-starved for 16-24 hours, and then either EGF (PeproTech House, London, England) or HRG-β 176-246 (R&D Systems, Inc., Minneapolis, MN) was added. Cells were incubated with growth factor for the indicated time-period, washed three times with phosphate buffered saline (PBS), and lysed with Bio-Plex lysis buffer (Bio-Rad Laboratories, Hercules, CA).

### qRT-PCR for screening of phenotype-specific transcriptional factors

For the qRT-PCR of 2,352 human transcription factors, we prepared gene-specific PCR primers based on identified mouse transcription factors as previously described [26]. PCR amplification was performed in triplicate using an ABI Prism 7900 HT instrument (Applied Biosystems, Lincoln Centre Drive Foster City, CA). The tailor-made reaction (20 μl) on the 384-well plates included 0.5 units of HotStar Taq DNA polymerase (QIAGEN, Hilden, Germany) and associated ×1 amplification buffer, 1 mM MgCl_2_, 160 μM dNTPs, 1/38 000 SYBR Green I (Molecular Probes, Carlsbad, CA), 7% DMSO, 0.4% ROX Reference Dye (Invitrogen, Carlsbad, CA), 300 nM of each primer, and 2 μl of 40-fold diluted first-stranded cDNA synthesis reaction mixture. The polymerase activation step at 95°C for 15 min was followed by 40 cycles of 15 sec at 94°C, 30 sec at 60°C, and 30 sec at 72°C. Dissociation curve analysis, which evaluates each PCR product to be amplified from single cDNA, was carried out in accordance with the manufacturer's protocol. Several PCR products were also checked by agarose gel electrophoresis (data not shown). For the qRT-PCR data analysis, differentially expressed genes in response to EGF and HRG stimulation were searched for by calculating the index *I*, defined as follows;

where *x*_*t*, *EGF *_and *x*_*t*, *HRG *_represent qRT-PCR-based expression levels of gene *x *after *t *hours administration of EGF and HRG, respectively. Those genes which displayed maximum expression levels of less than 300 were omitted prior to the index value calculation. The distribution of *I *was approximated by a normal distribution with mean and standard deviation values of -0.1 and 0.7, respectively. Those genes with *I *values greater than 3.0 were extracted as differentially expressed genes and their expression pattern was confirmed by GeneChip-based expression data analysis.

### Microarray analysis

Total RNA was isolated using TRIzol reagent (Invitrogen) and then purified using the QIAGEN RNeasy Mini kit. RNA quality was assessed using a Bioanalyzer (Agilent Technologies, Santa Clara, CA). First- and second-strand cDNA synthesis, biotin-labeled cRNA synthesis, fragmentation of cRNA and hybridization reactions were performed using a one cycle cDNA synthesis kit (Affymetrix, Santa Clara, CA). GeneChip (Affymetrix U133A 2.0 chip) experiments were carried out according to the manufacturer's protocol. Scanned images were processed using GeneChip Operating Software (GCOS) to determine the signal intensity of probe sets. Scaling was performed using the Single-Array Expression Analysis function in GCOS and the target value was set to 500. Microarray data used in this study was submitted to Gene Expression Omnibus database (GSE13009).

### Enrichment analysis

For each of the four transcription factors selected from the qRT-PCR results and confirmed by the microarray data, genes showing an HRG-induced expression pattern were correlated with these transcription factors (correlation coefficient ≥0.85) and those that showed higher expression with HRG than with EGF (*I *> 2.2) were extracted. As a result, 38, 35, 52 and 44 probe sets were selected for *EGR4*, *FOSL-1*, *FHL2 *and *DIPA*, respectively. The index value *I *was calculated for the microarray gene expression profile in the same way as for the qRT-PCR data. *I *= 2.2 corresponds to 95% point of fitted normal distribution. Probe Set IDs were converted to Entrez Gene IDs according to the manufacturer's annotation and then corresponding gene ontology (GO) terms were extracted from the ID mapping table provided by NCBI. Those probe sets without Gene ID were discarded in the analysis. Enrichment analysis was performed by Fisher's exact test followed by Bonferonni's correction. Those GO terms which showed a small *p*-value (*p *< 0.05) were regarded as enriched.

### qRT-PCR for gene expression validation

For the qRT-PCR of siRNA-transfected cells, 500 ng of total RNA was reverse transcribed using the PrimeScript RT reagent Kit (TaKaRa, Shiga, Japan). cDNA equivalent to 5 ng of total RNA was used for all the PCR reactions. The sequences of the primers are as follows; 5'-GCA CCG TCA AGG CTG AGA AC-3' and 5'-ATG GTG GTG AAG ACG CCA GT-3' for *GAPDH*, 5'-ACT TGA AAG CAT CCA TGT GTG TGG AC and 5'-GGC CTG GCT CAA CAT GCT ACT AA-3' for *c-FOS*, 5'-AGC AGC AGC AGG TGA TTG GA-3' and 5'-CGC AGA TCA GCT CAT CAC AGA AG-3' for *FOSL-1*, 5'-TGG CAT AAC GAC TGC TTT AAC TGT A-3' and 5'-GTG TGA GAT CAC AAG CAG CAA-3' for *FHL2*. All PCR reactions were performed using SYBR Premix Ex Taq (TaKaRa) or KAPA SYBR Fast kit (KAPA Biosystems, Cape Town, South Africa) in the Thermal Cycler Dice Real Time System TP800 (TaKaRa). qRT-PCR was performed in triplicate for each sample using default two-step amplification procedures in a 25 μl reaction volume according to the manufacturer's instructions. The conditions for the PCR reaction were as follows: 60°C for 30 sec, 95°C for 15 sec, followed by a maximum of 40 cycles of 95°C for 5 sec, and 60°C for 20 sec. The standard curve method was used to determine relative quantification of mRNA abundance [27]. For normalization of the qRT-PCR data, *GAPDH *expression was used as the control at each time point.

### Immunoblotting

Cell lysate was cleared by centrifugation, and the protein concentration of the supernatant was determined using a protein assay reagent (BioRad Laboratories). Protein phosphorylation levels and total proteins were analyzed by Western blotting as previously described [28] using the requisite antibodies. For the Western blot analysis, anti-ERK (p44/42 MAP kinase), anti-phospho-ERK (Thr202/Tyr204), and anti-alpha-tubulin antibodies were purchased from Cell Signaling Technology, Inc. (Danvers, MA). Anti-FOS, anti-FRA-1 and anti-EGR4 antibodies were purchased from Santa Cruz Biotechnology, Inc. (Santa Cruz, CA), anti-FHL2 antibody from AVIVA System Biology (San Diego, CA) or Santa Cruz Biotechnology, and anti-c-JUN antibody from Upstate (Billerica, MA). Protein band intensities were quantified using a densitometer (Fuji Film Corp., Tokyo, Japan).

For the detection of phosphorylated-ERK, cells were treated with 10 nM EGF or HRG at each indicated time point. Protein lysates were prepared using the Cell lysis kit (Bio-Rad Laboratories). The presence of phospho-ERK1/2 was detected using the Bio-Plex phospho-protein assay kit (Bio-Rad Laboratories) according to the manufacturer's protocol. Data from the reaction was acquired and analyzed using the Bio-Plex suspension array system (Luminex 100 system, Bio-Rad Laboratories). Total proteins for ERK were measured using the Bio-Plex total protein assay kit (Bio-Rad Laboratories).

### Gene silencing with RNAi

For 60-mm-diameter experiments, MCF-7 cells were harvested by trypsinization and seeded at 1.2 × 10^6 ^cells/well using HiperFect Transfection Reagent (QIAGEN) and CombiMAG magnetofection reagents (Chemicell GmbH, Berlin, Germany) according to manufacturer's protocol. For *c-FOS *siRNA transfection, 10 nM siGENOME SMARTpool *c-FOS *siRNA or non-targeting control siRNA (Dharmacon, Cramlington, UK) was included in the transfection mixture. For *FOSL-1 *and *FHL2 *siRNA transfection, siRNA was designed based on sequences specific for human cDNA; 5'-GCA TCA ACA CCA TGA GTG G-3' for *FOSL-1 *and 5'-GCA AGG ACT TGT CCT ACA A-3' for *FHL2*. Antisense and sense siRNA oligonucleotides with dTdT 3' overhangs were synthesized by TaKaRa Bio Inc. Each siRNA was added to 10 nM and compared with controls transfected using identical concentrations of control siRNA mixture. After 48 hrs of transfection, cells were starved for 16 hrs in serum-free DMEM. Cells were stimulated with 10 nM HRG for the indicated times in the figures, harvested and then lysed in preparation for qRT-PCR and immunoblotting.

### Protein-protein interaction assay

Cells were washed with PBS and lysed in the lysis buffer. The supernatant was recovered after centrifugation. For immunoprecipitation, 0.5 mg of the aforementioned supernatant protein was incubated with 6 μg of antibody and rotated overnight for 4°C. Then, 20 μl of protein G Plus/Protein A-agarose Suspension (Calbiochem, Madison, WI) was added and the mix incubated for 3 hrs at 4°C on a rotating platform. Following centrifugation, beads were washed three times with HNTG buffer (20 mM HEPES (pH 7.5), 150 mM NaCl, 10 mM NaF, 1 mM Na_3_VO_4_, 0.5 μg/mL leupeptin, 1 μg/mL pepstatin A and 0.2 mM PMSF). Bound proteins were eluted with SDS sample buffer, resolved by SDS-PAGE, and transferred onto PVDF membranes.

### Electrophoretic mobility shift assays (EMSAs)

Nuclear protein was extracted from ligand-treated MCF-7 cells. All procedures were carried out on ice. Treated cells were harvested, suspended in a five-fold of CH buffer (10 mM HEPES-KOH (pH 7.5), 2 mM MgCl_2_, 1 mM EDTA, 1 mM EGTA, 10 mM KCl, 1 mM DTT, 10 mM NaF, 0.1 mM Na_3_VO_4_, and 0.5 mM PMSF added just before use), incubated on ice for 10 min and then centrifuged. Precipitates were resuspended in a three-fold volume of CH buffer containing 0.2% NP-40 and then homogenized using a Dounce homogenizer (20 strokes). Homogenates were centrifuged at 15,000 rpm for 10 min at 4°C. To the supernatants was added 1 mL of CR buffer (40 mM HEPES-KOH (pH 7.5), 0.4 M KCl, 1 mM DTT, 10% (v/v) glycerol, 0.1 mM PMSF and 0.1% (w/v) aprotinin) and 5 M NaCl was added to a final concentration of 500 mM. Mixtures were incubated on ice for 30 min, centrifuged at 24,000 rpm for 20 min, and aliquots were frozen and stored at -80°C until use.

A double-stranded oligonucleotide corresponding to the 12-*O*-tetradecanoylphorbol-13-acetate (TPA) response element (TRE) binding site was synthesized with the following sequence; 5'-CTC TGG CAG GTG CGT CAG TCC G -3' for -318TRE [29]. Synthesized oligonucleotides were end-labeled with [γ-^32^P]ATP (Amersham Bioscience, Piscataway, NJ) and cleaned up using a Gel Shift Assay System (Promega, Madison, WI) according to the manufacturer's instructions. Briefly, 15 μg of nuclear extract was incubated in gel shift binding buffer containing ^32^P-labeled oligopeptides. For the supershift assay, the reaction mixture contained 2 μl of anti-FHL2 specific antibody. For the oligonucleotide competition experiments, the reaction mixture was preincubated with a 50-fold excess of unlabeled oligonucleotide probes prior to the addition of radioactive probes. Samples were resolved on 5% non-denaturing polyacrylamide gel and exposed by radioautography (Fuji Film Corp.).

## Results

We previously showed that stimulation of MCF-7 breast cancer cells with EGF and HRG resulted in very similar early transcription profiles up to 90 min, however subsequent cellular phenotypes differed [25]. Although both ligands evoked qualitatively similar signaling activities and early transcription, HRG induced prolonged signaling activities and significant expression of c-FOS protein, while EGF induced a transient signal and negligible amounts of c-FOS. Therefore, it was hypothesized that this all-or-none supply of c-FOS transcription factor, which plays a major role in AP-1 activation [14], might trigger changes in late transcription which determine ligand-specific cell fate. In an effort to investigate this hypothesis, MCF-7 cells were exposed to growth factors for longer time periods and the temporal expression of transcription factors was monitored.

### HRG and EGF induced different transcription factors

**Figure 1:**
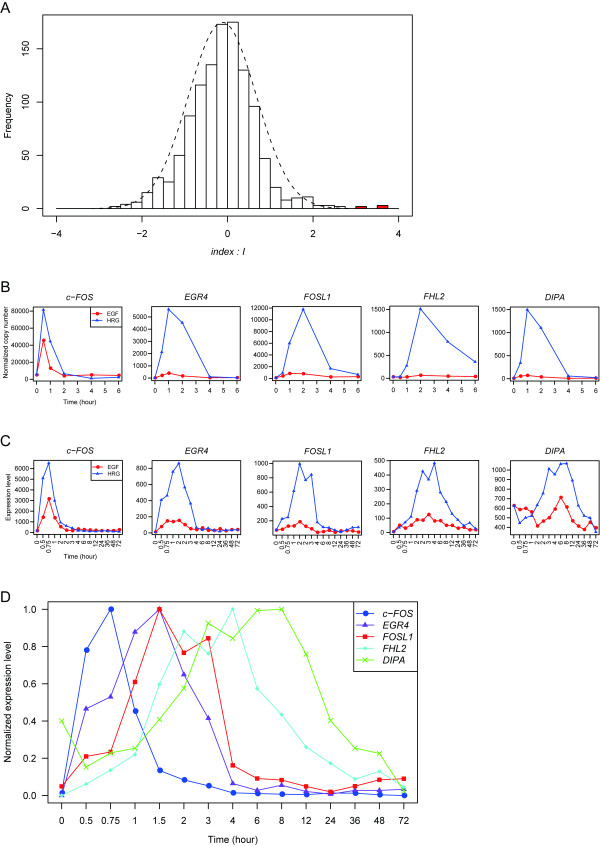
**Time-dependent gene expression of EGF- or HRG-induced transcription factors**. (A) Gene expression of 2,352 human transcription factors in EGF- and HRG-treated MCF-7 cells was measured by qRT-PCR. The index value *I *for these transcription factors was calculated and its distribution is shown. The fitted normal distribution is shown as a dotted curve. Red bars represent genes whose *I *value is greater than 3.0, with five transcription factors (*c-FOS*, *EGR4*, *FOSL-1*, *FHL2 *and *DIPA*) satisfying this criterion. (B-D) qRT-PCR and GeneChip gene expression analyses were performed using growth factor-treated MCF-7 cells. The expression of mRNA after treatment of the cells with 10 nM HRG or EGF was monitored by qRT-PCR for up to 6 hrs and for up to 72 hrs using a GeneChip microarray system. The expression profiles of five transcription factors with high *I *value are shown. (B) Gene expression time-course of five transcription factors measured by qRT-PCR. (C) Gene expression time-course of five transcription factors measured by GeneChip. (D) HRG-induced gene expression measured using a GeneChip microarray system. These five transcription factors show sequential peaks of mRNA expression.

Expression analysis of 2,352 human transcription factors following up to 6 hrs (0, 0.5, 1, 2, 4 and 6 hrs; 6 time points) exposure to HRG or EGF was performed using qRT-PCR and the expression profiles were investigated. The *I *value, a ratio representing the difference between HRG- and EGF-induced gene expression, was calculated as described in the Materials and Methods section. Fig. 1A shows the distribution of *I *values for the transcription factors targeted. The results revealed that HRG, unlike the case with EGF, uniquely induced significantly high expression levels of five transcription factors (I > 3.0) (Fig. 1A red bars), which were identified as *SUPT4H1*, *EGR4*, *FOSL-1*(FRA-1 gene), *FHL2 *and *DIPA *(also known as coiled-coil domain containing protein 85B) (Table 1). Surprisingly, neither EGF-specific nor significant EGF-induced expression of transcription factors was detected.

**Table 1:** HRG-induced genes identified by qRT-PCR

Gene Symbol	Gene Name
*SUPT4H1*	suppressor of Ty 4 homolog 1 (*S. cerevisiae*)
*DIPA*	hepatitis delta antigen-interacting protein A
*FHL2*	four and a half LIM domains 2
*FOSL1*	FOS-like antigen 1*

*FOSL-1 is the gene name of FRA-1 protein.

Having identified HRG-induced expression of specific transcription factors from the qRT-PCR results, gene expression analysis using microarray was performed in an effort to determine the time-course expression profile of the aforementioned five transcription factors and other genes up to 72 hrs (0, 0.5, 0.75, 1, 1.5, 2, 3, 4, 6, 8, 12, 24, 36, 48 and 72 hrs; 15 time points). Although results of the investigation of *SUPT4H1 *mRNA expression using a microarray platform were inconsistent with the results obtained from the qRT-PCR analysis (data not shown), results pertaining to the other four transcription factors (*EGR4*, *FOSL-1*, *FHL2 *and *DIPA*) showed good agreement with the qRT-PCR data, and time-dependent mRNA expression was only observed for HRG-stimulated cells (Fig. 1B and 1C). Interestingly, the expression peaks of these four transcription factors appeared one after the other following the expression of *c-FOS *(Fig. 1D). The gene expression data from both the microarray and qRT-PCR analysis showed that HRG could induce relatively higher expression levels of the *c-FOS *gene compared with EGF (ca. 60% of HRG-induced *c-FOS*), and that the ligand-induced differences in expression became more pronounced during the later expression of *EGR4, FOSL-1*, *FHL2 *and *DIPA *(Fig. 1B and 1C). Transient *c-FOS *expression might suggest negative regulation by other transcription factors. Furthermore, the data implies that *c-FOS *might be replaced in the AP-1 complex by another AP-1 member, thereby altering a function of AP-1 [30] to facilitate response to HRG-mediated cell fate.

The product of the first inducible gene, *EGR4*, is a zinc-finger protein, a member of the EGR family of transcription regulatory factors which plays a critical role in mediating enduring forms of neuronal plasticity and the regulation of inflammatory cytokine gene transcription [31]. EGR3 and EGR4 interact with NFκB p50 and p60 to activate transcription inflammatory-gene promoters [32]. FRA-1 (*FOSL-1 *protein) is a member of the AP-1 family of transcription factors that includes c-FOS, and plays an important role in cell motility, invasion, and maintenance and progression in several transformed and neoplastic cells [33-35]. FRA-1 is known to contribute to cellular differentiation processes through interactions with c-FOS [36] and requires signal-dependent protein stabilization through the DEF domain (FXFP or FXYP) in a manner similar to c-FOS [37-39]. FHL2 can serve as a transcriptional coactivator of AP-1, androgen receptor, CREB and CREM in transformed cells and is known to associate with JUN and FOS [40,41]. Interestingly, FHL2 has been reported to bind to phosphorylated ERK *in vitro *and inhibit nuclear localization and activity of ERK in stimulated cardiomyocytes, indicating its antagonistic role in cellular signaling [42]. DIPA is known as a partner of C/EBP β and was reported to have an inhibitory effect on adipocyte differentiation in preadipocytes [43].

**Figure 2:**
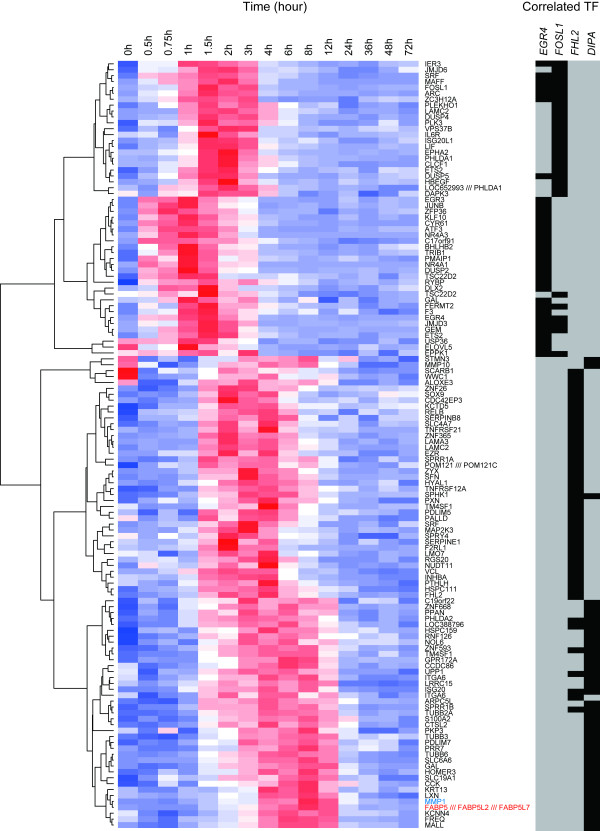
**Expression profiles of genes correlated to four transcription factors**. A hierarchical clustering was applied to the HRG-induced gene expression profiles of those genes correlated with the selected transcription factors (*EGR4*, *FOSL-1*, *FHL2 *and *DIPA*) and the results are shown as a heat map. If multiple probe sets with the same Gene ID were selected for the same transcription factor, a probe set which showed maximum *I *was selected in this analysis. Correlated transcription factors are highlighted in black in the right panel of the figure. Expression levels were normalized to yield mean and standard deviation values of 0 and 1, respectively, before applying the clustering algorithm. High and low expression levels are depicted in red and blue, respectively.

**Figure 3:**
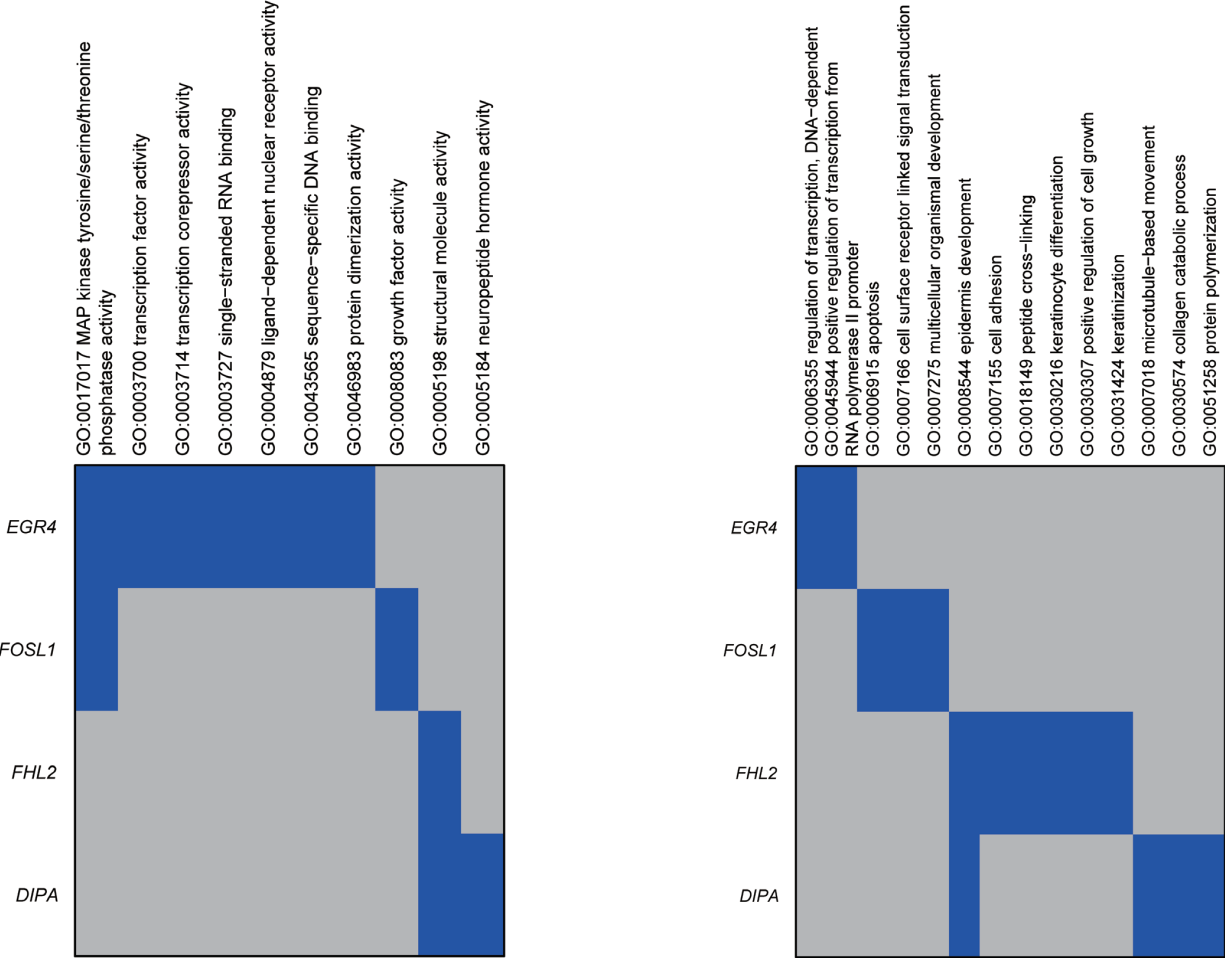
**Functions and pathways related to candidate transcription factors**. Enrichment analysis results utilizing molecular function (left panel) and biological process (right panel) terms for genes correlated with the four transcription factors (*EGR4*, *FOSL-1*, *FHL2 *and *DIPA*) and specifically induced by HRG are shown as a heat map. Enriched gene ontology terms are highlighted in blue.

We also identified genes (146 probe sets) the expression patterns of which correlated with the aforementioned candidate four transcription factors, and which showed higher expression following HRG stimulation compared with EGF, as determined from our microarray data (Fig. 2). Enriched transcription factors associated with the 146 probe sets which correspond to 126 genes were searched for using GATHER [44]. As a result, AP-1, SP3 and SRF were identified as enriched transcription factors, suggesting that these transcription factors are involved in the transcriptional regulation of the correlated genes. Enrichment analysis using Gene Ontology [45] for each correlated gene cluster revealed time-dependent activation of gene function and pathways specifically activated by HRG (Fig. 3). In the early phase, genes related to transcriptional regulation and the maintenance of signal transduction were activated, and then the expression diminished in the later phase. In the mid to late phase, genes related to development and differentiation were activated. Manual curation of the gene list and clustering results revealed that the expression pattern of DIPA was significantly correlated with the expression of FABP5 (fatty acid biding protein 5), a differentiation marker of adipocytes [46] (Fig 2, red symbol). Furthermore, the expression of FABP5 was correlated with the expression of lipid regulators such as fatty acid desaturase 3 and metastasis markers such as matrix-metallopeptidase 1 (MMP1, Fig 2, blue symbol) only in HRG-stimulated cells. These data indicate that cells are directed towards differentiation and/or transformation of MCF-7 cells during the late phase following HRG stimulation. Thus, the appearance of expression peaks associated with these transcription factors showed a physiologically meaningful sequential profile (Fig. 1D).

Although DIPA contains a basic region-leucine zipper DNA binding and dimerization domain, which has similarities to FRA family members [47], DIPA has never been reported to interact with the AP-1 complex, EGR4 or FHL2. Moreover, several of our preliminary experiments failed to show an interaction of DIPA with any of the AP-1 proteins (data not shown). Therefore, we decided to focus on validating the mutual regulation of the other three transcription factors (EGR4, FRA-1 and FHL2) together with c-FOS.

### HRG induced time-dependent expression of c-FOS but not of EGR4

**Figure 4:**
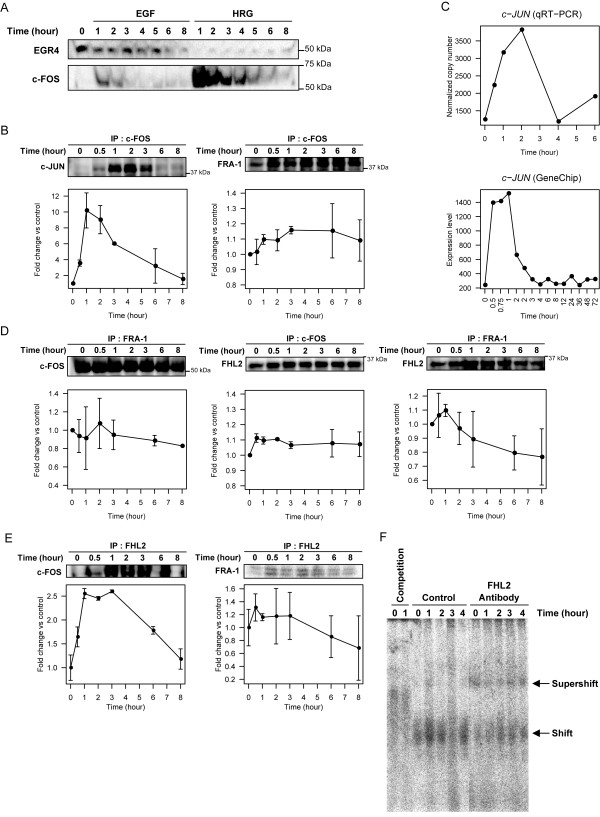
**HRG-induced expression of the candidate transcription factors**. (A) MCF-7 cells were treated with 10 nM HRG or EGF. Cell lysates were assayed for expression of EGR4 and c-FOS proteins using Western blot analysis. A representative figure of two independent experiments is shown. (B-D) HRG-induced expression of mRNA and protein associated with the AP-1 protein. HRG-treated cell lysates were immunoprecipitated using c-FOS antibody and subjected to Western blot analysis. Blots were then probed using an antibody against c-JUN or FRA-1. A representative figure of two independent experiments is shown. Mean values of quantified bands are shown with bars ± range (B). HRG-induced mRNA expression of *c-JUN *obtained by qRT-PCR (upper) and GeneChip (lower graph) analyses (C). HRG-treated MCF-7 cell lysates were immunoprecipitated using c-FOS, FRA-1 (D) or FHL2 (E) antibody. Immuno-complexes were detected using an antibody against c-FOS, FRA-1 or FHL2. The data are representative of two independent experiments. Mean values of quantified bands are shown with bars ± range. (F) EMSA analysis of FHL2 DNA-binding ability. ^32^P-labeled -318 TRE oligonucleotide probe, the sequence of which is present within the *FOSL-1 *promoter region, was incubated with HRG-treated cellular nuclear extract. For the competition assay, nuclear extracts were incubated with a 50-fold molar excess of unlabeled probe prior to use in the EMSA experiment. The -318 TRE sequence incubated with nuclear extract was observed in the control lanes and 'shift' bands indicate protein-nucleic acid complex formation. Supershift experiments of the complexes formed with the consensus -318 TRE sequence using FHL2 antibody.

In an effort to confirm the effects of HRG on EGR4 protein expression, a time-course treatment of MCF-7 cells with either EGF or HRG was performed. EGR4 protein was expressed at much higher levels in EGF-treated cells compared with HRG-treated cells (Fig. 4A), indicating that protein expression is not coordinated with gene expression in the case of EGR4. Furthermore, immunoprecipitation studies revealed that EGR4 clearly failed to interact with c-FOS or any other AP-1 proteins (data not shown), while c-JUN showed a strong interaction with c-FOS (Fig. 4B). EGR4 is a zinc-finger protein which binds the specific sequence GCGTGGGCG and negatively regulates transcription derived from its own gene promoter [31]. Neither *c-FOS *nor *FOSL-1 *contain the EGR4-binding sequence within their promoter regions (data not shown), suggesting that EGR4 could not interact with AP-1. A previous study reported that EGR4 can negatively regulate transcription derived from its own gene promoter whereas EGR1 can function as an activator [31]. EGR1 plays a role that contrasts that of EGR4, and is regulated by both MAPK-dependent and -independent pathways in PC12D cells [48], suggesting that EGR4 could also play a role in transcriptional regulation triggered by other pathways such as those involving PI3K or estrogen receptor signaling which are also active in MCF-7 cells [25,49]. However, based on our data, we concluded that EGR4 might not participate in the transcriptional regulatory network pertaining to cellular differentiation induced by HRG in MCF-7 cells.

### FRA-1 and FHL2 are associated with the c-FOS AP-1 complex

AP-1 proteins represent a group of IEG products that play important roles in triggering and regulating late transcription. IEG products which possess a DEF domain can act as sensors for ERK signaling [13,15,50]. Among our candidate transcription factors, FRA-1 and c-FOS possess DEF domains. In an effort to assay for AP-1 activity, a co-immunoprecipitation assay was performed (Fig 4B and 4D). The c-FOS and c-JUN gene expression profiles show good agreement with each other (Figs 1 and 4C), and c-JUN interacted with c-FOS immediately following HRG stimulation and then began to dissociate at 2 hr (Fig. 4B). On the other hand, the association between FRA-1 and c-FOS began to increase, and this interaction was maintained for several hours (Fig. 4D). Since c-FOS AP-1 family proteins cannot exist as homodimers after the dissociation of c-JUN at 2 hr, the results from Figs. 4B and 4D also indicate that FRA-1 co-immunoprecipitated with c-FOS (or *vice versa*) might associate with other binding partners, such as JUN AP-1 family proteins or FHL2. In Fig. 4D and 4E, FHL2 and c-FOS, and FRA-1 and FHL2 also showed a similar time-course in terms of complex formation. On the other hand, neither c-FOS nor FRA-1 could co-immunoprecipitate with JUNB and JUND (data not shown). Thus, results of the immunoprecipitation study suggested that c-FOS, FRA-1 and FHL2 might regulate the same transcriptional complex following regulation of the complex by c-JUN.

The upstream TPA response element (TRE) (-318) in the promoter region of *FOSL-1 *is important for inducing the *FOSL-1 *gene. TRE is a binding site for c-JUN and JUND [29,51] and is also targeted by c-FOS and FOSB [52], although *FOSL-1*transcription is also co-regulated by the binding of SRF, Elk1, ATF1 and CREB to the -276/-237 region for enhanced induction of *FOSL-1 *[51]. In an effort to determine whether FHL2 can bind to *FOSL-1 *DNA, EMSA using TRE oligonucleotides was performed (Fig. 4F). The mobility of the labeled -318 TRE probe shifted in the presence of nuclear extract (control) and was dependent on HRG stimulation. The addition of anti-FHL2 antibody to the binding reaction resulted in supershifts, suggesting that FHL2 could directly or indirectly bind to TRE (-318) on the *FOSL-1 *promoter and perhaps regulate the expression together with other AP-1 proteins.

### RNA interference of c-FOS suppressed FRA-1 and FHL2 gene and protein expression

**Figure 5:**
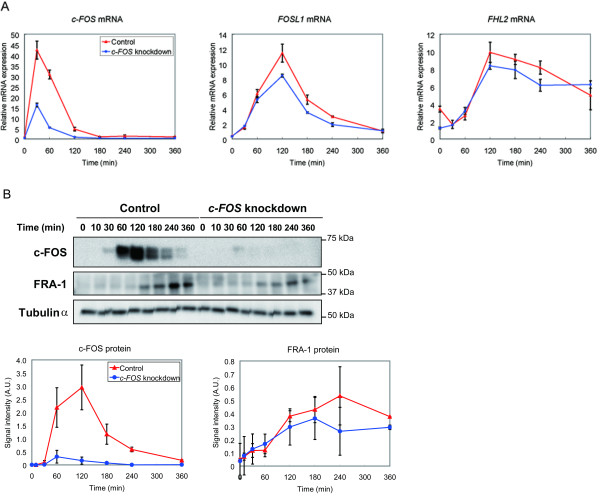
**Effect of *c-FOS *siRNA on *FRA-1 *and *FHL2 *expression**. (A) MCF-7 cells were treated with 10 nM HRG and mRNA expression was analyzed using qRT-PCR. Numbers on the *y*-axis represent the relative mRNA level; bars ± SEM. Data points were obtained from three replicas. *GAPDH *was used as a standard. (B) Western blot analysis. Each graph shows the densitometric analysis of blots obtained from two independent experiments. Protein signal intensity was determined by normalizing each value with tubulin-α protein. Bars ± range. Red lines, control cells; blue lines, *c-FOS *knockdown.

**Figure 6:**
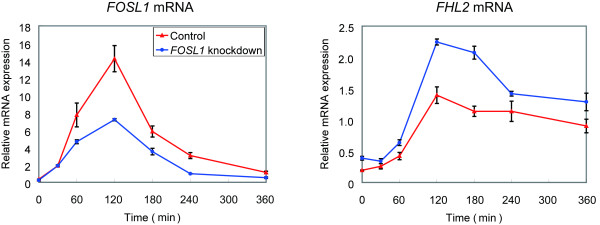
**Effect of *FOSL-1 *knockdown on *FHL2 *induction**. *FOSL-1 *and *FHL2 *expression levels following HRG (10 nM) treatment in MCF-7 cells interfered with *FOSL-1 *knockdown as determined by qRT-PCR analysis. Red lines, control cells; blue lines, *FOSL-1 *knockdown. Bars ± SEM.

If c-FOS regulates *FRA-1 *and *FHL2 *expression following HRG stimulation, the suppression of c-FOS could induce critical changes in late transcriptional control. When *c-FOS *gene expression was effectively suppressed using RNAi, *FOSL-1 *and *FHL2 *gene expression was also reduced in the presence of HRG (Fig. 5A). *c-FOS *knockdown also led to the suppression of FRA-1 protein expression (Fig. 5B), although FHL2 protein expression was not significantly reduced under the same conditions (data not shown). This indicates that both FRA-1 and FHL2 possibly act downstream to c-FOS. On the other hand, *FOSL1 *gene knockdown using RNAi induced an increase in *FHL2 *gene expression (Fig. 6).

**Figure 7:**
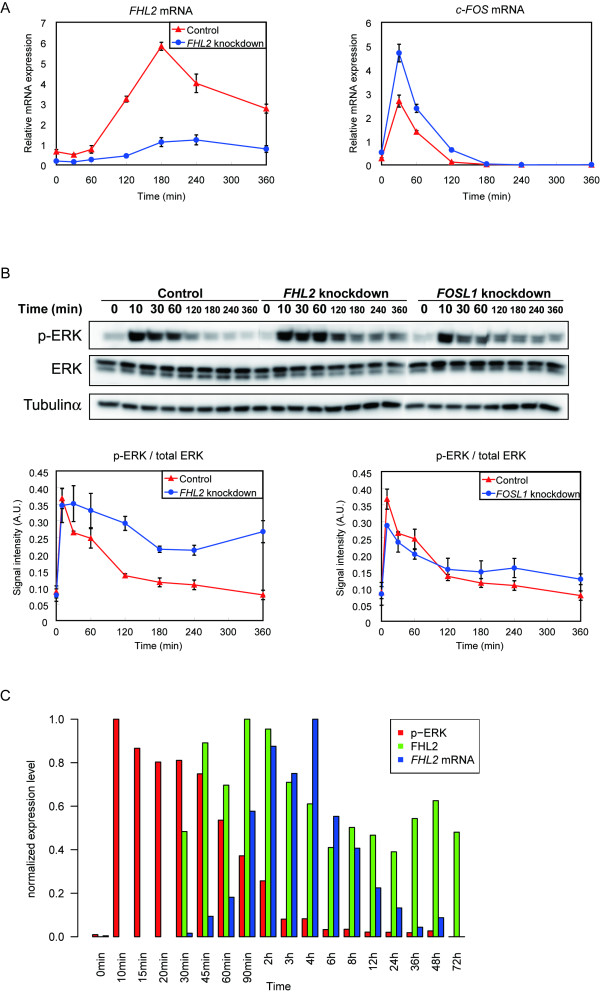
**Relationship among FHL2 and ERK activity**. (A) MCF-7 cells were transfected with *FHL2 *siRNA. *FHL2 *and *c-FOS *mRNA expression levels following 10 nM HRG treatment were analyzed by qRT-PCR. Bars ± SEM. (B) ERK phosphorylation and total protein levels were investigated by Western blot analysis. Phosphorylated protein expression levels were determined by normalizing each value with total protein. Mean and range values are indicated in the graphs. Two independent studies were performed for each experiment. Red lines, control cells; blue lines, *FOSL-1*/*FHL2 *knockdown. (C) Time-course appearance pf ERK phosphorylation and FHL2 protein and *FHL2 *mRNA expression. HRG-treated MCF-7 cells were lysed with Bio-Plex assay reagent. Normalized ERK phosphorylation is indicated by the red bars and FHL2 protein level determined by immunoblotting and *FHL2 *mRNA level as determined from the GeneChip data (from Fig. 1C) is indicated by the green and blue bars, respectively.

*FHL2 *knockdown caused an increase in *c-FOS *mRNA expression (Fig. 7A). This result is consistent with a previous report suggesting that FHL2 is a negative regulator of ERK [42], therefore ERK-regulated c-FOS expression is similarly negatively regulated by FHL2. Indeed, reduced gene expression of *FHL2 *resulted in the up-regulation of phospho-ERK (Fig. 7B). On the other hand, *FOSL-1 *gene knockdown resulted in a slight increase in ERK phosphorylation with little effect, which seems to be inconsistent with the result shown in Fig. 6. These results may suggest independent negative ERK regulation by *FOSL-1 *and *FHL2*, but not only by *FOSL-1*. In fact, the phosphorylation of ERK peaked within 10 min, was sustained for 1-2 hr, and then gradually decreased with the increase in *FHL2 *mRNA (Fig. 1B-C) and protein expression (Fig. 7C). These results indicated that both FRA-1 and FHL2 may negatively regulate the upstream signaling.

## Discussion

In MCF-7 breast cancer cells, HRG can elicit differentiation while treatment of cells with EGF leads to cell proliferation, processes which possess different intensities of signaling and gene regulation that work in a coordinated manner. Sustained activation of ERK by HRG resulted in stabilization of signaling mediators and changes in the supply of transcription factor proteins possessing an ERK sensing domain, thus leading cells along a path of irreversible differentiation [25]. Indeed, we identified a time-dependent interaction of several transcription factors whose expression levels were significantly elevated by HRG exposure, but not by EGF, during the course of a 72-hour time-course treatment of MCF-7 cells (Fig. 1). These transcription factors (Table 1) were expressed in a time-dependent manner, suggesting that they might regulate each other.

AP-1 consists of dimers of proteins encoded by *FOS *and *JUN *gene families, which have been widely implicated in differentiation, cell proliferation and transformation [53]. The FOS proteins (c-FOS, FOSB, FRA-1 and FRA-2) form heterodimers with the JUN protein families (c-JUN, JUNB and JUND) and regulate gene expression from TRE and TRE-like elements present within various promoters [29,38,54]. Our immunoprecipitation assay showed that the AP-1 complex in HRG-treated MCF-7 cells contained c-JUN, c-FOS and FRA-1, although the association of c-JUN in the complex is transient (1-3 hrs after HRG stimulation), while that of c-FOS and FRA-1 are sustained for up to 24 hr (our unpublished data). On the other hand, we demonstrated that FHL2 could associate with AP-1 proteins such as c-FOS and FRA-1. Taken together with those results, late transcriptional regulation in MCF-7 cells is mediated by a regulatory complex containing c-FOS, FRA-1 and FHL2. One possible scenario is that after c-JUN dissociates from the AP-1 complex, FHL2 could be a binding partner of c-FOS and/or FRA-1. In PC12 cells, nerve growth factor (NGF) induces sustained c-JUN and c-FOS activity in events leading to neural differentiation [39,55]. In MCF-7 cells, the gene expression profiles of *c-FOS *and *c-JUN *(Figs. 1 and 4C) induced by HRG were similar. Both expression patterns were transient for the first 1.5 hrs, and c-JUN seemed to dissociate from the complex within 3 hrs, even though c-FOS and FRA-1 remained within the complex for a longer period. *FRA-1 *gene expression was followed by the expression of two other AP-1 genes, *c-FOS *and *c-JUN *(Fig. 1), and FRA-1 protein induction was also prolonged for up to 6 hr following HRG stimulation (Fig. 5B). Thus, unlike NGF-induced signaling in PC12 cells, HRG treatment of MCF-7 cells evokes sustained formation of the AP-1 complex through FRA-1, but not c-JUN. Taken together, it seems that c-FOS and FRA-1, but not c-JUN, play an important role in MCF-7 cells in terms of late transcriptional processes and the progression of cell differentiation. Indeed, several pathological studies in breast cancer and neoplastic breast diseases have described FRA-1 as a useful marker in breast carcinogenesis, and its overexpression results in malignancy [56-59].

Moron *et al. *suggested that FHL2 may act as a coactivator of the c-FOS and c-JUN complex by stimulating AP-1-dependent transcription [41]. In fact, FHL2 was able to associate with the c-FOS and FRA-1 (Fig. 4D and 4E). The EMSA supershift experiment (Fig. 4F) also supported the view that FHL2 can bind to the -318 TRE sequence present within the *FOSL-1 *promoter region [51]. The -318 TRE region is important as a c-FOS responsive element for inducing enhanced expression of *FOSL-1 *[29,58] and serves as the binding site of AP-1 proteins such as c-JUN and JUND [59]. The result indicates that FHL2 may regulate the transcription of *FOSL-1 *through this region together with other AP-1 components. However, further investigations are required to delineate the interaction mechanism and regulation system involving FHL2 and AP-1 proteins. Furthermore, there appear to be conflicting reports concerning the role played by FHL2, where it has been suggested that FHL2 can serve as a repressor of MEK1-ERK1/2 signaling [60,61] or as an enhancer of the *c-FOS *promoter, whose gene is immediately expressed by activation of ERK1/2 [62,63]. In this study, we demonstrated using RNAi that *FHL2 *knockdown induced clear elevation of *c-FOS *gene expression and ERK activation. The gradual reduction in phospho-ERK levels was followed by an increase in *FHL2 *expression (Fig. 7C). These results suggested that FHL2 might inhibit both ERK activity and ERK-dependent transcription. On the other hand, *FOSL1 *knockdown induced elevated expression of *FHL2 *(Fig. 6), which suggests that FHL2 is not a downstream target of *FOSL1 *and negatively regulates ERK signaling independently from *c-FOS*-*FOSL1 *regulation, even though *FHL2 *induction seemed to be influenced by *c-FOS*.

Our analysis indicated that a time-dependent transcriptional regulatory network is vital in controlling the upstream ERK signaling pathway through a negative feedback loop pertaining to MCF-7 cell differentiation. However, there is a possibility that multiple signaling pathways such as those involving PI3K and the estrogen receptor could regulate transcription in and the differentiation of MCF-7 cells [64-66], and other signal-inducible factors could affect and interfere with the transcription factors we have not identified. Nevertheless, it is noteworthy that the transcription factors identified in the current analysis showed sequential time-dependent expression and mutual regulation, and that these functions are regulated by upstream ERK signals. However, later transcription inhibits the original upstream signal by negative feedback once the signal becomes unnecessary. In general, negative feedback functions to stabilize biological systems and several earlier studies have indicated the presence of negative feedback in cellular differentiation processes [67]. Our study has indicated that ligand-stimulated signaling activity is not only suppressed within intracellular signaling as demonstrated by EGF-induced Cbl down-regulation [68] or negative feedback within the MAPK cascade in PC12 cells [69], but is also regulated through *de novo *transcription.

## Conclusion

**Figure 8:**
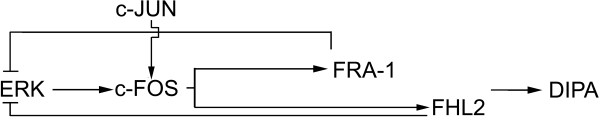
**Proposed scheme of timed transcriptional regulation for the HRG-induced differentiation model in MCF-7 cells**. Solid arrows and lines indicate the validated process in current issue and gray arrows are hypothetical process from the gene expression data in Fig. 1.

Fig. 8 depicts the suggested late-transcriptional regulatory network involved in the differentiation of MCF-7 cells. In early transcription, sustained ERK activation induces IEG expression including c-FOS. This DEF-domain containing protein, c-FOS, can be stabilized by the upstream ERK signal. This expression is followed by expression of another AP-1 family member, FRA-1, which then triggers FHL2 expression. FRA-1 protein requires stabilization by ERK to function [70], and therefore ERK activity is required until such time as FRA-1 and c-FOS proteins are stabilized. The sequential appearance of FHL2 can then switch off unnecessary ERK activity, which results in complete signal cut-off. The differentiation process in MCF-7 cells could therefore be regulated by negative feedback control from the transcriptional network.

## Authors' contributions

YS, KI and NY carried out qRT-PCR, immunoassays and microarray experiments for acquisition of data. TE and TN performed analysis and interpretation of gene expression data. TT, HS, YH, MO and YS obtained funding, conceived, designed and supervised the study. YS, TN, and MO drafted the manuscript. All authors read and approved the final manuscript.
